# The Transcriptional Landscape of *BRAF* Wild Type Metastatic Melanoma: A Pilot Study

**DOI:** 10.3390/ijms23136898

**Published:** 2022-06-21

**Authors:** Elena Lastraioli, Federico Alessandro Ruffinatti, Giacomo Bagni, Luca Visentin, Francesco di Costanzo, Luca Munaron, Annarosa Arcangeli

**Affiliations:** 1 Department of Experimental and Clinical Medicine, University of Florence, Viale GB Morgagni 50, 50134 Florence, Italy; elena.lastraioli@unifi.it (E.L.); giacomo.bagni@unifi.it (G.B.); 2 Department of Life Sciences and Systems Biology, University of Torino, Via Accademia Albertina 13, 10123 Torino, Italy; federicoalessandro.ruffinatti@unito.it (F.A.R.); luca.visentin@unito.it (L.V.); luca.munaron@unito.it (L.M.); 3 Medical Oncology Unit, Azienda Ospedaliero-Universitaria Careggi, Largo Brambilla 3, 50134 Florence, Italy; dicostanzofrancesco@tiscali.it; 4 Complex Dynamics Study Centre (CSDC), University of Florence, 50100 Florence, Italy

**Keywords:** metastatic melanoma, wild type *BRAF*, transcriptomics, microdissection, ribosomes

## Abstract

Melanoma is a relatively rare disease worldwide; nevertheless, it has a great relevance in some countries, such as in Europe. In order to shed some light upon the transcriptional profile of skin melanoma, we compared the gene expression of six independent tumours (all progressed towards metastatic disease and with wild type *BRAF*) to the expression profile of non-dysplastic melanocytes (considered as a healthy control) in a pilot study. Paraffin-embedded samples were manually micro-dissected to obtain enriched samples, and then, RNA was extracted and analysed through a microarray-based approach. An exhaustive bioinformatics analysis was performed to identify differentially expressed transcripts between the two groups, as well as enriched functional terms. Overall, 50 up- and 19 downregulated transcripts were found to be significantly changed in the tumour compared to the control tissue. Among the upregulated transcripts, the majority belonged to the immune response group and to the proteasome, while most of the downregulated genes were related to cytosolic ribosomes. A Gene Set Enrichment Analysis (GSEA), along with the RNA-Seq data retrieved from the TCGA/GTEx databases, confirmed the general trend of downregulation affecting cytoribosome proteins. In contrast, transcripts coding for mitoribosome proteins showed the opposite trend.

## 1. Introduction

According to the definition reported by the Dictionary of Cancer Terms (https://www.cancer.gov/publications/dictionaries/cancer-terms, accessed on 22 February 2022), melanoma is “A form of cancer that begins in melanocytes (cells that make the pigment melanin). It may begin in a mole (skin melanoma), but can also begin in other pigmented tissues, such as in the eye or in the intestines”.

Overall, analysing the global incidence and mortality worldwide, melanoma is a relatively rare disease that affected 325,000 people in 2020, and 57,000 died because of the disease (source: Globocan 2020, https://gco.iarc.fr/today/online-analysis-table, accessed on 18 December 2021). Nevertheless, skin melanoma has a great relevance in certain countries, such as Australia and Europe, where the incidence rate is higher. In Europe, skin melanoma represents the seventh-most frequent malignancy and accounts for roughly 46% of the incident cases in the whole world (https://gco.iarc.fr/today/data/factsheets/cancers/16-Melanoma-of-skin-fact-sheet, accessed on 18 December 2021). Moreover, it has been shown that some complex diseases, such as diabetes mellitus, might represent a risk factor for melanoma occurrence [1], and Endothelial Progenitor Cells (EPCs) are known to be selectively recruited within the tumour [2] and might represent a potential tool for therapy in this group of patients, especially the younger ones in which the EPC levels are higher [3].

Melanocytes can give rise to benign lesions called melanocytic naevi that can progress towards malignant lesions termed melanomas. Melanomas are classified according to the TNM staging system (AJCC staging manual 8th edition, issued in 2016 and updated in 2018) [4], and a global melanoma database has been released [5]. The data obtained from the clinical and pathological evaluations of melanoma are combined to divide patients into staging groups with different outcomes [6]. In addition, specific classification systems for melanoma were defined by Clark [7] and Breslow [8] long ago. The expression profiles and somatic mutations of advanced lesions and metastases have been defined and are reported in the Cancer Genome Atlas Network [9], while less is known about the initial phases of melanoma progression [10]. The most frequent genetic alteration described in melanoma is *BRAF* mutation, present in roughly 50% of the patients, according to the COSMIC database (Catalogue Of Somatic Mutations In Cancer) [11]. The vast majority of *BRAF* mutations are represented by a missense mutation named V600E, leading to the substitution of glutamic acid with a valine in codon 600 [9,12]. The final phenotype is characterised by the constitutive activation of the mitogen-activated protein kinase (MAPK) pathway, sustaining cell proliferation and preventing apoptosis. BRAF inhibitors (such as Vemurafenib and Dabrafenib) have been approved for the treatment of metastatic melanoma [13], since they significantly improve progression-free and overall survival, although resistance is rapidly acquired [14]. Currently, for patients not carrying *BRAF* mutations, no target therapy is available; therefore, a significant effort is needed to better define their molecular profile in order to identify potential molecular markers and targets for therapy. Therapy for this group of patients mainly relies on immunotherapy and checkpoint inhibitors [15,16,17], and searching for potential predictive biomarkers in response to biological agents is warranted in melanoma, as well as in other metastatic tumours [18]. 

The aim of this pilot study was to analyse the transcriptomic profile of patients suffering from metastatic melanoma without *BRAF* mutations in order to evaluate the possible differences in the expression profiles between advanced melanoma cells and non-dysplastic melanocytes serving as the healthy control.

## 2. Results

### 2.1. RNA Extraction and Array Hybridisation

**Figure 1:**
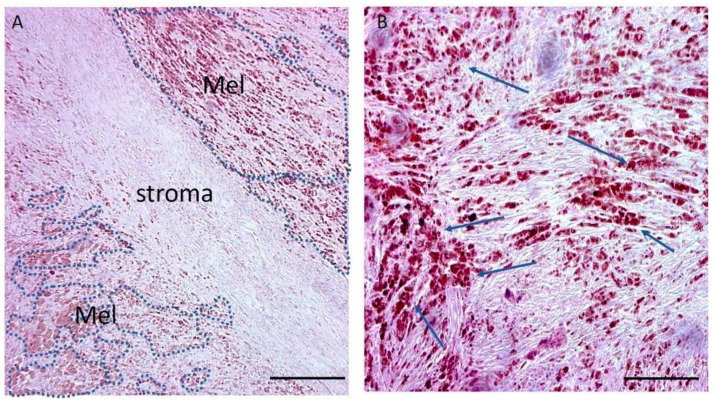
**Histopathological microphotograph of a representative melanoma sample**. Haematoxylin–eosin staining of a representative melanoma sample showing areas rich in brown-coloured melanin-producing tumour cells (Mel, dashed blue shapes and blue arrows). (**A**) The brown-coloured areas rich in melanocytes are clearly identified by the presence of melanin that is absent in the central portion of the slide (composed of stromal tissue). Scale bar: 200 µm. (**B**) The higher magnification of this microphotograph allows the observation of melanin-rich cells (indicated by the arrows) and gives information about the cell pleomorphism within the tumour. Scale bar: 50 µm.

In order to obtain a comparative transcriptomic profile of melanoma cells relative to the healthy tissue (i.e., non-dysplastic naevi composed of healthy melanocytes representing the normal counterpart of melanoma cells), a pilot study was designed, and we performed a manual microdissection of paraffin-embedded surgical samples [19] of both normal tissue and *BRAF* wild type melanoma from patients whose clinicopathological characteristics are in Table 1. The samples then enriched in the melanocytic population (Figure 1) were processed for RNA extraction and then hybridised on Agilent arrays after RNA validation (see the details in Section 4, Supplementary Figure S1).

### 2.2. Differential Expression Analysis

The gene expression data from the microarray experiments were pre-processed according to a standard pipeline, as described in Section 4. The so-obtained normalised log_2_ expression data were then filtered and subjected to a differential expression analysis (DEA) using the rank product statistics (see Section 4 for details). In particular, *n* = 6 independent samples of *BRAF* wild type metastatic melanoma were compared to a reference array representing a pool of healthy tissues enriched with non-dysplastic melanocytes (biological average of *n* = 4 independent samples). Transcripts featuring a BH-FDR *q*-value < 0.05 and a |log_2_FC| > 0.5 were deemed as differentially expressed genes (DEGs). Overall, the DEA returned a list of 84 statistically significant probes, but only 69 of them could be annotated. Specifically, 50 up- and 19 downregulated transcripts were found to be significantly changed in the tumour compared to the control tissue, as reported in Table 2 and Table 3, respectively.

In order to further strengthen the transcriptomic analysis results, the differential expression of six DEGs (*APOE*, *GAPDH*, *ACTB*, *RNA28S1N5*, *RPL31*, and *RPS17*), selected on the basis of the most relevant fold changes and *p*-values emerging from the microarray experiments, was further assessed by RT-qPCR in a small subset of melanoma samples from the microarray analysis cohort (as described in the relative Section 4). Considering a *p*-value ≤ 0.05 to assess the significance, four of the six selected DEGs (*ACTB*, *RPS17*, *RPL31*, and *RN28S1N5*) showed statistically significant differences between the melanoma samples and healthy melanocytes, with fold change directions (expressed as relative expression fold changes performed by the ΔΔCT method) consistent with the microarray screening results (Supplementary Figure S2). *GAPDH* and *APOE* differential expression failed to reach statistical significance when analysed by RT-qPCR in the same sample subset but still maintained the fold change consistency with the microarray results (Supplementary Figure S2).

### 2.3. Genes Related to Antigen Processing and Presentation Are Upregulated in Tumour vs. Control

**Figure 2:**
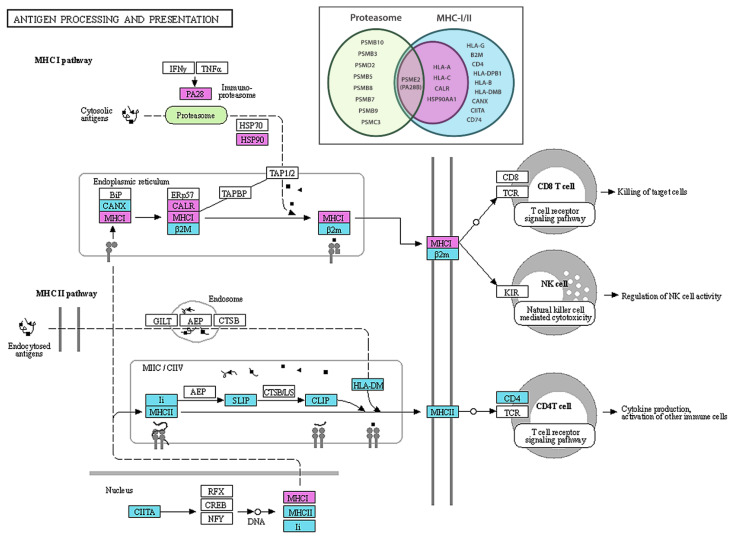
**Illustration of the antigen processing and presentation KEGG pathway**. Upregulated DEGs detected by rank product statistics are filled with magenta (*HLA-A* and *HLA-C* are here collectively referred to as *MHCI*; *HPS90* is a short for *HSP90AA1*; and *PA28* is an alias for *PSME1-2-3*). In cyan are the elements of the pathway additionally detected by the GSEA leading edge analysis. The GSEA also revealed a significant involvement of the proteasome complex (in green). Using the same colour code, the Venn diagram in the upper inset shows the complete lists of the official gene symbols found to be upregulated within the two KEGG pathways. The KEGG pathway map is hsa04612-antigen processing and presentation—Homo sapiens (human), modified and published with permission from Kanehisa Laboratories as the copyright holder.

DEGs that were found to be upregulated in metastatic melanoma samples compared to non-dysplastic controls were tested for functional enrichment using the ToppFun web tool (https://toppgene.cchmc.org/, accessed on 22 February 2022). The full table of the statistically significant terms retrieved from such a query can be found as Table S1 in the Supplementary Materials section. Inspecting the results, it is noticeable how the top-most ranked functional terms and pathways were almost all related to the positive regulation of some features of the immune response, involving 32 out of the 50 upregulated DEGs resulting from the DEA. For example, the most relevant GO terms referring to biological processes (BPs) were *innate immune response*, *defense response to other organism*, *regulation of immune system process*, *cell activation*, *response to external biotic stimulus*, *leukocyte mediated immunity*, and *antigen processing and presentation of exogenous peptide antigen via MHC class I* (BH-FDR < 1.7 × 10^−4^). Accordingly, the most significant KEGG pathway [20,21] was *antigen processing and presentation* (BH-FDR = 6.4 × 10^−4^), accounting for five DEGs having a central role in the MHC class I pathway: *HLA-A*, *HLA-C*, *CALR*, *PSME2*, and *HSP90AA1* (Figure 2, genes in magenta).

**Figure 3:**
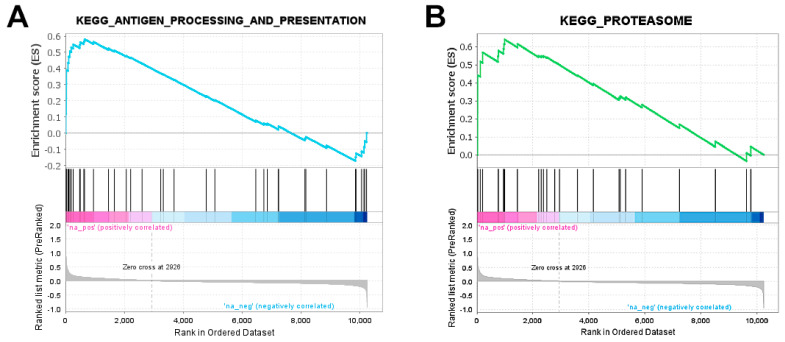
**GSEA enrichment plots**. Profile of the running ES score (upper boxes), and positions of the gene set members on the rank-ordered list from the microarray experiments (lower boxes) for (**A**) the antigen processing and presentation and (**B**) proteasome KEGG pathways, respectively. The leading edge comprises that portion of the gene set between the (absolute) ES maximum and the nearest edge of the ranked list.

Such a finding was confirmed by the GSEA (see Section 4), according to which, the gene set corresponding to this pathway was positively enriched (NES = 2.03, FDR *q*-value = 0.064, Figure 3A). More in detail, the leading edge analysis identified 13 main genes involved in the upregulation of both the MHC class I and class II pathways, thus extending the previous set of five DEGs detected on the basis of gene-wise hypothesis testing (Figure 2, genes in magenta plus genes in cyan). In addition, the GSEA pointed at a significant positive regulation of the *proteasome* complex (NES = 2.07, FDR *q*-value = 0.069, Figure 3B), another KEGG pathway term deeply connected to the previous one, with a leading edge featuring nine genes coding for different proteasome subunits, including pivotal proteasome activator subunit 2 (*PSME2*) as a linker between the two gene sets (Figure 2, green ellipse and Venn diagram).

### 2.4. Cytosolic Ribosome Proteins Are Downregulated in Tumour vs. Control

Strikingly, the vast majority of the transcripts found to be downregulated in metastatic melanoma compared to the control reference (i.e., non-dysplastic melanocytes) were related to cytosolic ribosomes (see Table 3). More in detail, 13 out of the 19 downregulated DEGs corresponded to ribosomal proteins (rProteins) of either the large (60S) or the small (40S) cytosolic ribosome subunit. In addition, two different probes targeting the product of the *RNA28SN5* gene—the ribosomal RNA, giving rise to the 28S subunit—were among the 19 DEGs featured by the list of downregulated genes.

**Figure 4:**
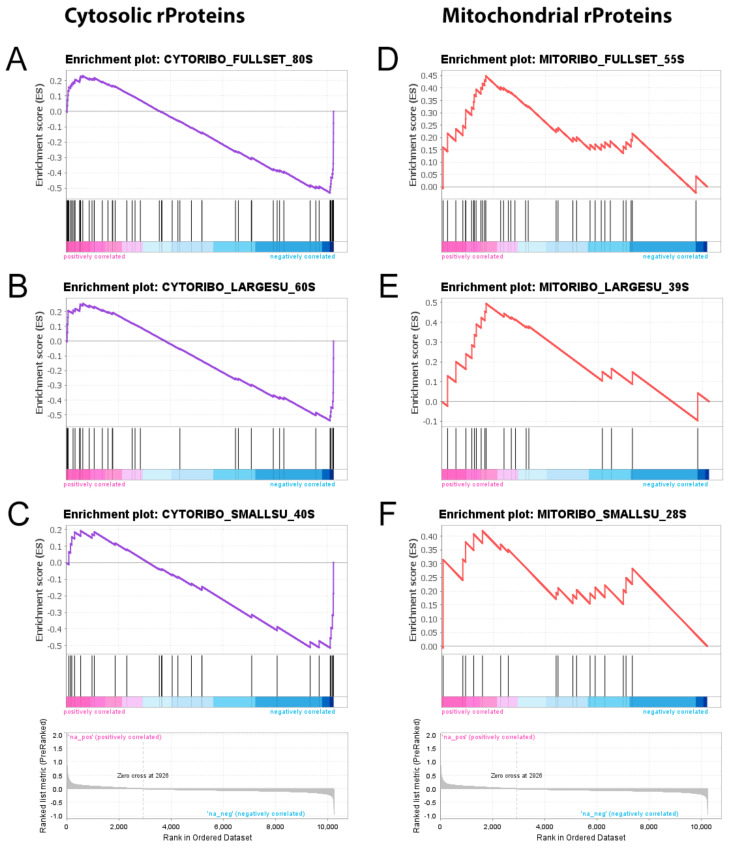
**Gene set enrichment analysis of the ribosomal protein gene set**. ((**A**)–(**C**)) Downregulated cytosolic rProtein transcripts were significantly enriched (*q*-values: 1.5 · 10^−4^, 0.002, and 0.013 for the 80S, 60S, and 40S subunit gene sets, respectively). ((**D**)–(**F**)) In contrast, the mitochondrial rProtein genes showed a consistent upregulation (*q*-values: 0.205, 0.151, and 0.231 for the 55S, 39S, and 28S subunit gene sets, respectively).

In order to confirm and extend these results, we ran a GSEA, testing the whole spectrum of rProteins of both cytosolic and mitochondrial origin. To do this, we took advantage of the already available Ribosomal Protein Gene Set (RPGS), which is the complete list of all human gene symbols related to ribosomes we assembled for a recent work in order to answer a similar scientific question [19]. Such an analysis confirmed a significant downregulation of the structural constituents of both the large (60S) and the small (40S) cytosolic ribosome subunits (Figure 4A–C). On the contrary, and most interestingly, mitochondrial rProteins did not show any significant downregulation but, rather, an opposite trend (Figure 4D–F).

### 2.5. External Validation through TCGA vs. GTEx Cohorts

In order to rule out any technical artefact related to microarray hybridisation or the sample origin, we decided to externally validate these findings using the UCSC Xena Browser (University of California, Santa Cruz, CA, USA, http://xena.ucsc.edu/, accessed on 24 February 2022) that provides a convenient way to access gene expression data stored in TCGA database for the comparative analysis of tumour samples with the normal analogies available from GTEx database (https://gtexportal.org/home/, accessed on 24 February 2022) [22,23]. TCGA samples were thus filtered based on cancer type (Skin Cutaneous Melanoma, SKCM), stage (metastatic), and genomic subtype (*BRAF* wild type). The so-obtained cohort featured 179 SKCM samples that were compared with the corresponding healthy GTEx cohort of normal skin tissue made out of 557 samples for a total sample size of *n* = 736.

**Figure 5:**
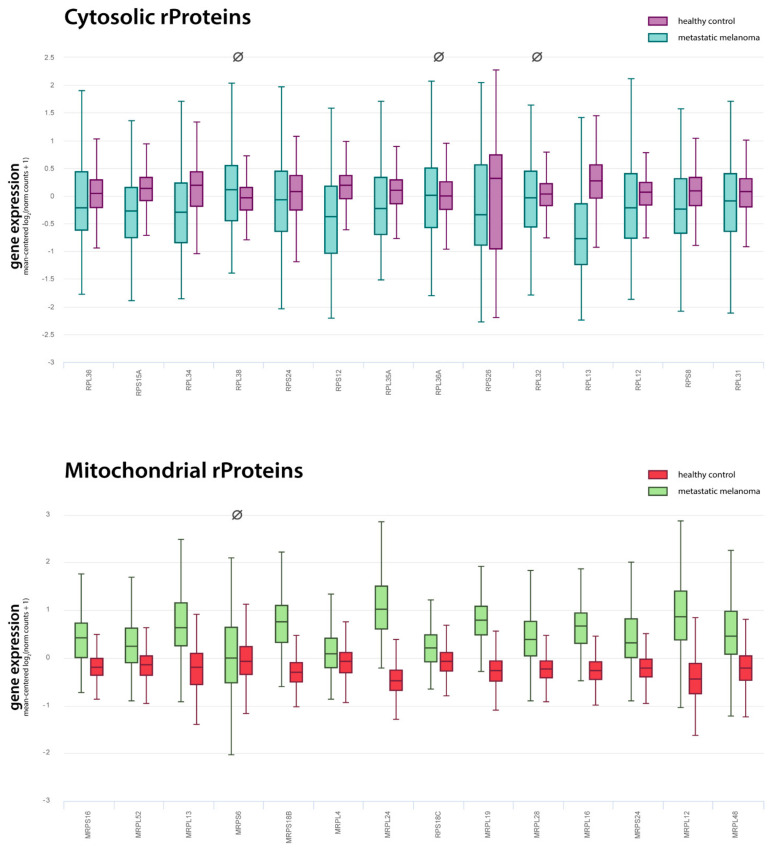
**Gene expression for ribosomal proteins from TCGA/GTEx databases for metastatic melanoma samples**. The Xena browser was used to filter TCGA samples and keep only the data from Skin Cutaneous Melanoma studies of the metastatic type and with no mutations in the BRAF gene. The final cohort featured 179 tumour samples from TCGA and 557 healthy samples from GTEx, for a total sample size of *n* = 736. The RNA-Seq expression data are given in units of log_2_ RSEM normalised counts, gene-wise mean-centred, and shown as boxplots for the 14 cytosolic (magenta and teal) and the 14 mitochondrial (red and green) rProteins that emerged from the leading edge analysis of microarray data. Overall, 24 out of 28 differential expressions were confirmed by TCGA data, supporting the evidence of a generalised downregulation of the cytosolic rProteins and an overexpression of the mitochondrial ones. The four unconfirmed comparisons are marked with the symbol Ø (see Table 2 for more details).

A thorough validation was carried out for the following sets of genes resulting from the corresponding GSEA leading edge analysis shown in Figure 3 and Figure 4: MHC pathway (14 genes), proteasome (9 genes), cytosolic rProteins (14 genes), and mitochondrial rProteins (14 genes). Notably, almost all differential expressions we tested could be confirmed by TCGA/GTEx RNA-Seq data in terms of both the change direction (log_2_FC sign) and statistical significance (Figure 5). The detailed validation scores are given in Table 4.

**Figure 6:**
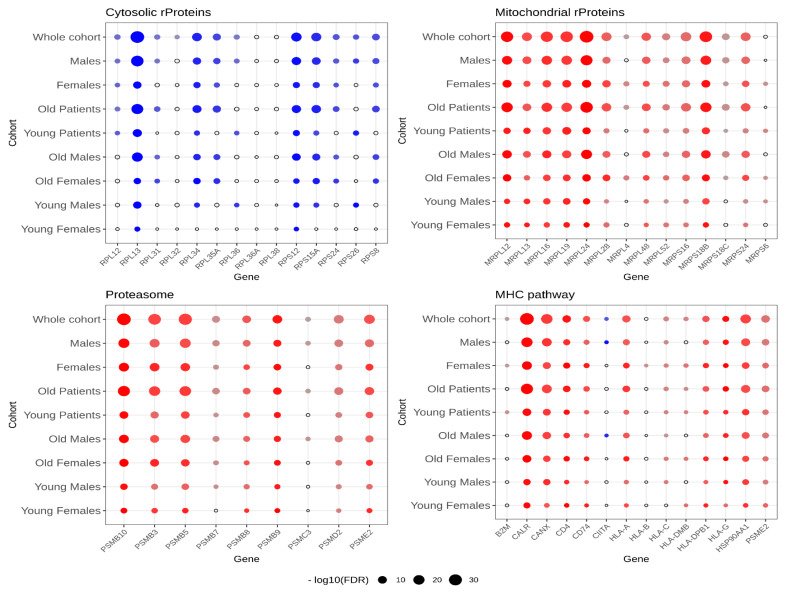
**Differential gene expression between healthy GTEx samples and cancer TCGA samples**. The four gene sets under investigation are shown in different panels. The overall cohort was divided into nine sub-cohorts: all samples, only males or females, only old (>50 years of age) or young, and combinations of sex and age. The size of each dot is proportional to the −log_10_(FDR) score for each statistical test. The colour and colour intensity of each point are proportional to the fold change of that comparison: red dots for upregulated genes (in tumour compared to healthy tissues), and blue dots for downregulated genes. Both colour and point size scales are independent for each gene panel. The lower legend is indicative of the overall trend of the data.

To control for the possible effects from the age and sex of the patients, these two covariates were also considered after downloading the specific metadata from the consortium portals (see Section 4). The results of such an analysis are presented as dot plots in Figure 6 and numerically in Supplementary Table S2 for the four gene sets of interest separately. Even if the overall dysregulation patterns could be substantially confirmed in all sub-cohorts, age appeared to be an important exacerbating factor (compare the Old Patients with Young Patients rows in the four panels of Figure 6). On the contrary, sex did not seem to be a discriminating factor, except, perhaps, in the downregulation of cytosolic rProteins, which were almost unaltered in the sub-cohort of Young Females (upper-left panel in Figure 6).

## 3. Discussion

In the pilot study reported in the present paper, the RNA extracted from paraffin-embedded samples was hybridised on Agilent microarrays to assess the transcriptomic profile of *BRAF* wild type metastatic melanoma compared to the transcriptional reference of non-dysplastic melanocytes serving as the healthy control. Beyond the canonical DEA, followed by the functional enrichment analysis of the resulting DEGs, the GSEA was extensively used both to deepen the involvement of some relevant pathways of interest (i.e., MHC and proteasome) and to quantify the overall dysregulation of the whole rProtein spectrum. In addition, because of the limited sample size of this pilot study, and due to the particular nature of the RNA starting material, all our findings were validated querying the TCGA/GTEx gene expression databases to consider larger cohorts of patients, and at the same time, the hallmark gene sets from MSigDB were tested through a dedicated GSEA to check the consistency and the reliability of the expression levels, as measured by our microarray experiment (see below and Supplementary Table S3).

The GSEA and ToppFun functional enrichment analysis of the upregulated DEGs showed a consistent involvement of the immune system, with 70% of the overexpressed genes coherently annotated to some immune response-related process. This is in line with the well-known immunogenic nature of melanoma and the recent literature pointing at MHC-I/II protein expression as a powerful prognostic marker to predict the effectiveness of anti-CTLA-4 and anti-PD-1 immunotherapy in metastatic melanoma and other cancer types [24,25,26,27,28]. Even though most of these papers agree on the fact that a transcriptional downregulation of the MHC-I and MHC-II genes is a common feature of advanced untreated melanomas, in our study, the opposite seems to be true. Notably, this cannot be ascribed to some spurious effects induced by drugs—such as antibodies targeting the immune checkpoints—since all the samples we used for RNA extraction were excised from the patients before any therapeutic schedule. Moreover, microarray technical validation performed via RT-qPCR in a sample subset (Supplementary Figure S2) further corroborated the reliability of the microarray data presented here. In addition, even the RNA-Seq data from the TCGA/GTEx databases confirmed such a significant overexpression of all the MHC class I genes (*HLA-A*, *HLA-B*, and *HLA-C*), as well as the MHC class II (*HLA-DMA*, *HLA-DOA*, *HLA-DPA1*, *HLA-DQA1*, and *HLA-DRA*), in *BRAF*-wild type metastatic melanoma compared to healthy skin tissue (Supplementary Figure S3). Rather, fold-change signs could be dependent on the particular stage at which melanoma samples were collected. Indeed, the MHC gene expression profile has already been reported to be heavily dependent on tumour progression, and its gradual loss is likely to facilitate the evasion of cancer cells from immune surveillance [29].

The other gene set we found to be upregulated in our cohort of metastatic melanoma patients compared to healthy controls was related to proteasomal function. Beyond its increased expression, the proteasome complex in melanoma cells may also be overactive because of the overexpressed *PSME2* gene and the proteasome activator complex subunit 2 (aka *PA28B*), thus contributing, in turn, to the increased antigen presentation by the MHC class I pathway discussed above (see Figure 2). These data agree with the notion that melanoma cells heavily rely on proteasomal function to survive, so that selective proteasome inhibitors have already been used as new attractive therapeutics for this type of cancer [30,31,32].

On the other hand, the DEGs downregulated in the melanoma samples compared to the healthy controls were mostly related to cytosolic ribosomal proteins (rProteins). This was not completely unexpected given the accumulating evidence that relates cancer onset and progression with alterations of cell translational machinery [33]. Specifically, both enhanced and reduced ribosome biogenesis and protein synthesis have been reported to be associated with cancer in mammals, depending on the particular type of tissue and stage taken into account [34,35,36,37,38]. For this reason, rProteins configuration in metastatic melanoma was evaluated more in depth by running a GSEA of all the structural constituents of both cytosolic and mitochondrial ribosomes. Interestingly, the two ribosome types showed opposite patterns of deregulation: while cytosolic rProteins tended to be under-expressed in metastatic melanoma, the mitochondrial ones were sharply upregulated.

Such a finding is of great interest considering, in particular, the data we recently published in another paper addressing transcriptional alterations in colorectal carcinoma [19]. As in the present case, even in that study, we were able to find a consistent change in the rProtein expression but with a different FC sign, in that the upregulation of rProteins concerned cytosolic ribosomes and not the mitochondrial ones. Importantly, in both studies, all the ribosomal transcriptional alterations we reported found confirmation in the TCGA/GTEx large cohorts of patients.

As a final note on the DEG lists emerged from our analysis, it is worth noting that the set of significantly dysregulated genes was obtained through the hard thresholding (cut-off 0.05) of the whole transcriptome genes sorted by increasing *q*-values, with an additional cut-off on the fold change (|log_2_FC| > 0.5), as reported in Section 4. While this conservative approach is effective in reducing the number of false-positive hits (i.e., controlling for type I error), the statistical power may be affected, resulting in an increased number of false negatives, especially in the case of small sample sizes and RNA partial degradation (as in the case of paraffin-embedded samples). For this reason, it is not surprising that the DEG lists lack some genes whose dysregulation is expected in metastatic melanoma. Nevertheless, when the GSEA was performed testing the “Hallmark gene sets” (the *H*-collection of the MSigDB) and the weighted contribution from all the log_2_FC-ranked genes of the array was taken into account, many significant hallmark gene sets emerged related to: proliferation processes (*MYC_TARGETS_V1* and *P53_PATHWAY*); cancer (*mTORC1_SIGNALING*, *EPITHELIAL_MESENCHIMAL_TRANSITION*, and *UNFOLDED_PROTEIN_RESPONSE*); the immune system response (*COMPLEMENT*, *ALLOGRAFT_REJECTION*, and *INTERFERON_GAMMA_RESPONSE*); and alteration of the metabolism (*GLYCOLYSIS* and *OXIDATIVE_PHOSPHORYLATION*). This is in excellent agreement with the results of other GSEAs performed on several different melanoma datasets (see, e.g., [39]), ultimately confirming the reliability of the gene expression profile returned by our microarray experiments (see Supplementary Table S3 for a complete list of the significant Hallmark Gene Sets, as returned by the GSEA).

As for the energy metabolic pathways involved in the early phases of melanoma pathogenesis, the key process is represented by glycolysis, and after the occurrence of *BRAF* mutations, the stimulation of transcription factors acting as key regulators of such process makes it even more effective [40,41]. Moreover, in *BRAF*-mutated cells, the Oxidative Phosphorylation (OXPHOS) is inhibited [42]. It is well-known that, between these two metabolic phenotypes, a dynamic switch occurs, and plasticity plays a key role in melanoma [43,44,45], leading to metabolic reprogramming of the cells. To make the picture more complex, it has been shown that some melanomas are able to exploit diverse nutrients and energy sources to adapt to different extracellular conditions, thus showing a “hybrid” glycolysis/OXPHOS metabolic phenotype [46,47,48]. Finally, the so-called “Reverse Warburg” effect has been described in melanoma cells [45,47,49]. This effect relies on the stimulation of cancer-associated fibroblasts (CAFs) that increase their glucose upload and lactate secretion through Monocarboxylate Transporter (MCT) family proteins [50]. Moreover, lactate can be internalised by cancer cells via MCT and conveyed into the Krebs cycle, thus fuelling OXPHOS. In this view, immune cells can deregulate metabolic pathways, representing a link between the deregulated pathways that emerged in this paper. As a further confirmation, the transcript of *SLC66A1*, encoding an MCT, was upregulated in our cohort (see Table 2).

All the proteins encoded by mitochondrial DNA are involved in the assembly and functioning of the respiratory complexes, along with the proteins encoded by nuclear DNA. For this reason, the OXPHOS biogenesis is subjected to a synchronised regulation of the mitochondrial and cytoplasmic ribosomes. Considering that—in contrast to the mitochondrial rRNA—mitochondrial rProteins are synthesised in the cytosol after the translation of mRNA of nuclear origin, the interplay between the nuclear and mitochondrial components for ribosome production and the consequent synthesis of the various proteins involved in glycolysis, and OXPHOS is extremely complex. Since our data derive from a transcriptomic approach, they cannot give insights on the expression and function of the glycolytic and OXPHOS enzymes; therefore, no robust hypothesis on functional significance can be proposed. Nevertheless, these data could pave the road for further evaluations in which biochemical and physiological assays, together with proteomic and metabolomic approaches, can be used to define the activity and expression levels of the key glycolytic/OXPHOS enzymes. 

The results reported in this paper might be relevant for two main reasons: (i) the central role of protein synthesis and energy metabolism in cancer and (ii) the fact that, despite the many recent reports about cytosolic ribosome aberrant function in cancer, there are still few data about the 55S mitochondrial counterparts and their functional interplay with 80S ribosomes. For example, the evidence of possible mitoribosome onco-patterns could provide a new rationale for the design (or repurposing) of novel antibiotics specific for cancer treatment, a still-debated clinical practice [51].

Taken together, our data point at a complete and deep remodelling of protein synthesis and degradation in metastatic melanoma that suggests, alongside biopsy genotyping, a more integrated evaluation of specific gene expression patterns—in particular, those related to MHC, proteasomes, and rProteins—as a practice that could help in choosing the most effective treatment in a context of personalised medicine.

## 4. Materials and Methods

### 4.1. Patients

Six patients (2 females, 4 males with mean age at diagnosis of 60.3 years, range 46–70) suffering from metastatic melanoma not harbouring *BRAF* mutations were enrolled for the study between April 2016 and October 2018 within the OMITERC study coordinated by Medical Oncology Unit, Azienda Ospedaliero-Universitaria Careggi (Florence). All the patients provided informed written consent, and the study was approved by the local Ethical Committee of Azienda Ospedaliero-Universitaria Careggi (BIO.16.028, released on 5 October 2016). Paraffin-embedded samples of the primary tumours were retrieved from the archives of the Department of Medical Biotechnologies, University of Siena, Italy. The clinical and pathological features of the patients were defined by experienced medical oncologists and pathologists according to the relevant guidelines (Table 1). Moreover, 4 non-dysplastic naevi were also collected from the same institution as above.

### 4.2. Sample Preparation

In order to obtain tumour-enriched samples, paraffin-embedded specimens were manually micro-dissected, applying the same protocol as in [15]. Briefly, 20-µm-thick sections were cut, put on no positively charged slides, and counterstained with Meyer’s Haematoxylin following the standard protocol. In order to achieve the enrichment of the tumour component of the metastatic melanoma samples, tumour areas were identified by an experienced operator (EL), collected through a sterile needle, and transferred to a fresh Eppendorf tube for further processing. 

### 4.3. RNA Extraction and Quality Control

The total RNA was extracted from the enriched samples with the AllPrepDNA/RNA FFPE kit (Qiagen, Hilden, Germany), according to the manufacturer’s protocols. The extracted RNA was then checked for its quality and integrity by the Agilent 2100 Bioanalyzer with the RNA 6000 Nano kit (Agilent Technologies, Santa Clara, CA, USA). The RNA concentration was also measured by a Nanodrop ND-1000 (Thermo Scientific, Waltham, MA, USA).

### 4.4. Microarray Hybridisation

A one-color microarray-based gene expression analysis was applied to analyse the RNA samples on the Agilent-026652 Whole Human Genome Microarray 4 × 44 K v2 platform (Agilent Technologies, Santa Clara, CA, USA), according to the manufacturer’s protocols. To scan the microarrays, an Agilent G49000 DA SureScan Microarray scanner (Agilent Technologies, Santa Clara, CA, USA) was used, and subsequently, the data were extracted by Agilent Feature Extraction (Agilent Technologies, Santa Clara, CA, USA).

### 4.5. Differential Expression Analysis

Raw data obtained from microarray scanning were processed using Bioconductor software packages in the R environment. Briefly, fluorescence intensities were background-subtracted, log_2_-transformed, and quantile–quantile normalised to get the gene expression. Based on the results of hierarchical clustering and a PCA on the samples, one array (ID *Melanoma_5*) was excluded from the subsequent steps of the analysis. Low-intensity probes (featuring a log_2_ expression below 6.3 in more than one melanoma sample) were filtered out of the expression matrix as probes targeting unexpressed genes. Overall, 13,455 probes out of 34,127 (~40%) were retained at the end of the filtering procedure, and their log_2_ expression values were tested for differential expression using rank product statistics. In particular, *n* = 5 melanoma biological replicates were compared against the single reference represented by the healthy biological mRNA pool of *n* = 4 non-dysplastic naevi (RankProd v3.18.0 Bioconductor package, one-sample design) [52,53,54,55,56]. *p*-values were adjusted for multiple comparisons, and all genes with a *q*-value (Benjamini–Hochberg False Discovery Rate, BH-FDR) < 0.05 were deemed as differentially expressed genes (DEGs) [57]. Finally, an additional cut-off on the fold changes (FCs) was applied to expunge from the DEG lists genes with a |log_2_FC| < 0.5.

### 4.6. Enrichment Analysis

The ToppFun web tool (by ToppGene Suite, https://toppgene.cchmc.org/, accessed on 22 February 2022) was used to analyse the DEG lists for functional enrichment through a hypergeometric hypothesis test [58]. All terms with a BH-FDR *q*-value < 0.05 were considered statistically significant. A Gene Set Enrichment Analysis was performed using GSEA v4.2.2 with the MSigDB database v7.5.1 (updated January 2022) [58,59]. Expression data from the microarray experiments were provided in the form of a pre-ranked list of genes (log_2_FC metric). Probes were collapsed into unique gene symbols before the analysis, and a standard (weighted) enrichment statistic was chosen. The Normalized Enriched Score (NES) and BH-FDR *q*-values are reported in the main text or Supplementary Materials for each gene set of interest. Within the context of the GSEA, the threshold of the *q*-value for a gene set to be considered statistically significant was set at 0.25. To evaluate the global transcriptional alterations affecting ribosomal proteins (rProteins), a custom gene set including all rProtein and rRNA genes was used. Details about such a custom Ribosomal Protein Gene Set (RPGS) have already been provided elsewhere [19].

### 4.7. TCGA/GTEx Validation

To provide an external validation of our main findings regarding the MHC pathway, proteasome, and rProteins, we used the UCSC Xena Browser (University of California, Santa Cruz, http://xena.ucsc.edu/, accessed on 24 February 2022) [22], which allows the direct comparison of tumour expression data stored in the database of The Cancer Genome Atlas (TCGA) consortium with healthy samples from the Genotype-Tissue Expression (GTEx) project database (https://gtexportal.org/home/, accessed on 24 February 2022) [23]. Specifically, we filtered TCGA data in order to keep samples only from the Skin Cutaneous Melanoma (SKCM) study of the metastatic type (TM, excised from patients not harbouring any *BRAF* mutation. As for the control group, all the normal skin samples retrieved from GTEx could be used. This led to a final comparison between *n* = 179 tumour samples and *n* = 557 normal tissues. This final cohort of *n* = 736 patients was then further characterised to take into account the contribution of age and sex as covariates. Namely, metadata for the TCGA consortium was downloaded with an ad hoc pipeline (https://github.com/MrHedmad/Edmund, accessed on 21 May 2022), while GTEx data was retrieved directly from the project’s data portal. Using these metadata, the samples were divided into nine categories: all samples (regardless of metadata), biological male patients, biological female patients, old patients (defined as greater than 50 years of age at the cancer diagnosis or sample acquisition for the TCGA and GTEx samples, respectively), young patients (defined as not old), young male patients, young female patients, old male patients, and old female patients. For each sub-cohort, the expression of the genes of interest (see Table 4 and Figure 6) was compared between the cancer (TCGA) and healthy (GTEx) samples. A comparison was performed with the Student’s *t*-test, double-sided, and the groupwise family error rate was corrected with the Benjamini–Hochberg procedure.

### 4.8. Real-Time PCR Validation

In order to further validate the transcriptomic data, 6 DEGs were selected according to the log_2_FC and *p*-values (see Table 2 and Table 3) for further validation by RT-qPCR. The selected gene expressions were assessed by using commercially available KiCqStart SYBR Green Primer pairs (Merck Millipore, Burlington, MA, USA), following the manufacturer’s protocols. Relative expression quantification was performed by the ΔΔCT method [60] using the gene expression normalisation approach identified in [61] for the melanoma samples. Gene expression was assessed in a small melanoma sample subset (*n* = 3) from the microarray cohort compared with the healthy control primary epidermal melanocytes.

## Figures and Tables

**Table 1:** Demographic and clinical features of the patients enrolled in the study.

Feature		Number (Percentage)
Age (mean, range)		60.3 (range 46–70)
Gender	Male	4 (66.7)
	Female	2 (33.3)
TNM stage at diagnosis	I	1 (16.7)
	II	2 (33.3)
	III	2 (33.3)
	IV	1 (16.7)
Metastatic site	Skin	3 (50.0)
	Lung	3 (50.0)
Histology	Nodular	2 (33.3)
	Superficial spreading	1 (16.7)
	Desmoplastic	2 (33.3)
	Naevoid	1 (16.7)
Clark’s level	1	0 (0.0)
	2	0 (0.0)
	3	0 (0.0)
	4	5 (83.3)
	5	0 (0.0)
	undefined	1 (16.7)
Breslow’s depth	I	0 (0.0)
	II	0 (0.0)
	III	5 (83.3)
	IV	1 (16.7)
Ulceration	No	5 (83.3)
	Yes	1 (16.7)
Regression	No	3 (50.0)
	Yes	1 (16.7)
	Undefined	2 (33.3)
Vascular involvement	No	2 (33.3)
	Yes	3 (50.0)
	Undefined	1 (16.7)
Perineural involvement	No	3 (50.0)
	Yes	1 (16.7)
	Undefined	2 (33.3)
*NRAS* status	Wild type	1 (16.7)
	Mutated	2 (33.3)
	Undefined	3 (50.0)
Best response	CR	0 (0.0)
	PR	3 (50.0)
	SD	1 (16.7)
	PD	1 (16.7)
	Undefined	1 (16.7)

**Table 2:** Upregulated genes, as resulted from the statistical comparison of melanoma vs. healthy samples (One Class Rank Product). Positive log_2_FC values indicate overexpression in the tumour compared to healthy tissue.

Probe ID	Gene Symbol	Description	log_2_FC	*q*-Value BH-FDR	*p*-Value
A_32_P137939	ACTB	actin beta	1.295	9.18 × 10^−8^	5.46 × 10^−11^
A_33_P3223592	APOE	apolipoprotein E	1.903	2.36 × 10^−9^	3.51 × 10^−13^
A_33_P3378531	AS3MT	arsenite methyltransferase	1.071	9.02 × 10^−6^	1.07 × 10^−8^
A_33_P3296198	C5orf63	chromosome 5 open reading frame 63	1.375	3.40 × 10^−6^	2.53 × 10^−9^
A_33_P3292854	CALR	calreticulin	0.642	4.77 × 10^−4^	1.74 × 10^−6^
A_33_P3280066	CAVIN1	caveolae associated protein 1	0.918	2.92 × 10^−5^	5.63 × 10^−8^
A_33_P3284508	CD14	CD14 molecule	1.829	4.27 × 10^−9^	1.27 × 10^−12^
A_33_P3229196	CD151	CD151 molecule (Raph blood group)	0.633	5.93 × 10^−4^	2.38 × 10^−6^
A_33_P3252612	CYP2W1	cytochrome P450 family 2 subfamily W member 1	0.546	1.77 × 10^−3^	9.09 × 10^−6^
A_24_P100673	EMC4	ER membrane protein complex subunit 4	0.676	4.38 × 10^−3^	2.83 × 10^−5^
A_33_P3333455	EMILIN1	elastin microfibril interfacer 1	0.535	3.99 × 10^−4^	1.31 × 10^−6^
A_33_P3379436	FAM74A4	family with sequence similarity 74 member A4	0.847	3.36 × 10^−5^	6.99 × 10^−8^
A_32_P342064	FTH1	ferritin heavy chain 1	0.505	5.50 × 10^−3^	3.80 × 10^−5^
A_23_P13899	GAPDH	glyceraldehyde-3-phosphate dehydrogenase	0.613	3.96 × 10^−4^	1.33 × 10^−6^
A_33_P3585268	GNAI2	G protein subunit alpha i2	1.332	1.01 × 10^−7^	5.25 × 10^−11^
A_24_P108451	GPI	glucose-6-phosphate isomerase	0.834	5.52 × 10^−6^	4.51 × 10^−9^
A_33_P3354322	GPX1	glutathione peroxidase 1	0.841	4.53 × 10^−5^	1.04 × 10^−7^
A_33_P3287218	GSTK1	glutathione S-transferase kappa 1	0.588	2.01 × 10^−4^	5.36 × 10^−7^
A_33_P3379962	HLA-A	major histocompatibility complex, class I, A	0.863	1.30 × 10^−3^	6.17 × 10^−6^
A_33_P3424803	HLA-C	major histocompatibility complex, class I, C	0.764	1.06 × 10^−3^	4.73 × 10^−6^
A_23_P162874	HSP90AA1	heat shock protein 90 alpha family class A member 1	0.616	3.78 × 10^−4^	1.21 × 10^−6^
A_23_P72737	IFITM1	interferon induced transmembrane protein 1	0.807	5.98 × 10^−6^	5.77 × 10^−9^
A_24_P605563	IGLC1	immunoglobulin lambda constant 1	0.517	5.77 × 10^−4^	2.23 × 10^−6^
A_23_P167168	JCHAIN	joining chain of multimeric IgA and IgM	0.529	2.26 × 10^−3^	1.22 × 10^−5^
A_32_P452655	LGALS9C	galectin 9C	0.605	1.38 × 10^−4^	3.58 × 10^−7^
A_23_P91619	MIF	macrophage migration inhibitory factor	0.957	9.65 × 10^−6^	1.22 × 10^−8^
A_23_P1904	MS4A2	membrane spanning 4-domains A2	0.630	2.62 × 10^−5^	4.47 × 10^−8^
A_23_P106844	MT2A	metallothionein 2A	0.552	4.39 × 10^−4^	1.53 × 10^−6^
A_33_P3239879	NAA38	N-alpha-acetyltransferase 38, NatC auxiliary subunit	0.782	4.66 × 10^−5^	1.04 × 10^−7^
A_23_P33022	POLR2L	RNA polymerase II, I and III subunit L	0.837	1.24 × 10^−5^	1.75 × 10^−8^
A_33_P3377199	PRDX1	peroxiredoxin 1	0.723	3.41 × 10^−4^	1.07 × 10^−6^
A_33_P3234899	PSMB3	proteasome 20S subunit beta 3	0.794	9.37 × 10^−5^	2.30 × 10^−7^
A_23_P65427	PSME2	proteasome activator subunit 2	0.701	1.03 × 10^−5^	1.38 × 10^−8^
A_23_P434301	PTMA	prothymosin alpha	0.582	4.56 × 10^−4^	1.63 × 10^−6^
A_33_P3382595	RN7SK	RNA component of 7SK nuclear ribonucleoprotein	0.680	2.80 × 10^−5^	4.37 × 10^−8^
A_23_P69431	RPL4	ribosomal protein L4	0.509	9.25 × 10^−4^	4.06 × 10^−6^
A_23_P106708	RPS2	ribosomal protein S2	0.664	4.08 × 10^−3^	2.46 × 10^−5^
A_23_P372874	S100A13	S100 calcium binding protein A13	1.043	2.04 × 10^−5^	3.04 × 10^−8^
A_24_P261169	SEMA4D	semaphorin 4D	0.574	3.85 × 10^−5^	8.30 × 10^−8^
A_33_P3413989	SERPING1	serpin family G member 1	1.286	6.05 × 10^−7^	4.05 × 10^−10^
A_23_P95213	SFTPC	surfactant protein C	0.854	5.55 × 10^−3^	3.87 × 10^−5^
A_33_P3481987	SLC16A12	solute carrier family 16 member 12	0.850	3.45 × 10^−05^	6.93 × 10^−8^
A_33_P3388491	SLC66A1	solute carrier family 66 member 1	1.440	6.62 × 10^−6^	7.38 × 10^−9^
A_33_P3587376	SNAR-A3	small NF90 (ILF3) associated RNA A3	1.423	8.95 × 10^−5^	2.13 × 10^−7^
A_33_P3370461	SUZ12P1	SUZ12 pseudogene 1	0.916	3.03 × 10^−5^	5.63 × 10^−8^
A_33_P3332690	SUZ12P1	SUZ12 pseudogene 1	0.597	2.65 × 10^−4^	7.69 × 10^−7^
A_33_P3274199	TP53I13	tumor protein p53 inducible protein 13	0.579	4.75 × 10^−4^	1.76 × 10^−6^
A_23_P325654	TRIM42	tripartite motif containing 42	0.925	4.70 × 10^−2^	9.40 × 10^−4^
A_33_P3409062	TYROBP	transmembrane immune signaling adaptor TYROBP	1.452	6.77 × 10^−8^	2.52 × 10^−11^
A_24_P101391	YBX1	Y-box binding protein 1	0.714	2.99 × 10^−4^	9.10 × 10^−7^

**Table 3:** Downregulated genes, as resulted from the statistical comparison of melanoma vs. healthy samples (One Class Rank Product). Negative log_2_FC values indicate downregulation in the tumour compared to healthy tissue.

Probe ID	Gene Symbol	Description	log_2_FC	*q*-Value BH-FDR	*p*-Value
A_23_P114445	MAGEE1	MAGE family member E1	−0.508	5.47 × 10^−5^	1.30 × 10^−7^
A_23_P112774	PTP4A3	protein tyrosine phosphatase 4A3	−0.513	2.71 × 10^−5^	4.23 × 10^−8^
A_33_P3332348	RN7SL1	RNA component of signal recognition particle 7SL1	−0.803	2.86 × 10^−6^	4.25 × 10^−10^
A_33_P3244165	RNA28SN5	RNA, 28S ribosomal N5	−1.378	6.92 × 10^−6^	2.06 × 10^−9^
A_33_P3346552	RNA28SN5	RNA, 28S ribosomal N5	−1.012	2.05 × 10^−5^	2.89 × 10^−8^
A_33_P3279708	RNU2−2P	RNA, U2 small nuclear 2, pseudogene	−0.954	7.28 × 10^−7^	5.41 × 10^−11^
A_23_P217068	RPL12	ribosomal protein L12	−0.753	5.96 × 10^−4^	3.32 × 10^−6^
A_24_P142228	RPL13	ribosomal protein L13	−0.578	2.01 × 10^−6^	4.48 × 10^−10^
A_32_P184518	RPL21	ribosomal protein L21	−0.807	6.08 × 10^−6^	3.62 × 10^−9^
A_32_P118258	RPL21	ribosomal protein L21	−0.861	2.75 × 10^−5^	4.09 × 10^−8^
A_24_P213783	RPL31	ribosomal protein L31	−0.944	6.65 × 10^−6^	4.45 × 10^−9^
A_23_P18142	RPL32	ribosomal protein L32	−0.568	1.53 × 10^−3^	1.20 × 10^−5^
A_33_P3329916	RPL6	ribosomal protein L6	−0.803	7.19 × 10^−6^	3.21 × 10^−9^
A_32_P857658	RPLP1	ribosomal protein lateral stalk subunit P1	−0.735	3.44 × 10^−4^	1.48 × 10^−6^
A_23_P147888	RPLP2	ribosomal protein lateral stalk subunit P2	−0.512	9.10 × 10^−4^	6.02 × 10^−6^
A_24_P418418	RPS17	ribosomal protein S17	−0.931	1.74 × 10^−5^	2.07 × 10^−8^
A_23_P116694	RPS26	ribosomal protein S26	−0.539	1.76 × 10^−5^	1.96 × 10^−8^
A_33_P3221680	RPS28	ribosomal protein S28	−0.750	1.48 × 10^−5^	1.43 × 10^−8^
A_23_P46182	RPS8	ribosomal protein S8	−0.757	9.37 × 10^−5^	2.86 × 10^−7^

**Table 4:** Number of genes subjected to validation by the TCGA/GTEx databases and their outcomes.

Pathway Name	GSEA Leading Edge	Xena Opposite FC	Xena Not Significant	Xena Concordant	Validation Score
MHC pathway	14	1	0	13	92.9%
proteasome	9	0	0	9	100%
cytosolic rProteins	14	1	2	11	78.6%
mitochondrial rProteins	14	0	1	13	92.9%

## Data Availability

Data are available upon request.

## Supplementary Materials

The following are available online at https://www.mdpi.com/article/10.3390/ijms23136898/s1.
