# Supplementary material for: The Transcriptional Landscape of BRAF Wild Type Metastatic Melanoma: A Pilot Study
Source: Int J Mol Sci. 2022 Jun 21;23(13):6898. doi: 10.3390/ijms23136898 (PMC9266837; doi:10.3390/ijms23136898)
Supplement: Supplementary file 1 [file ijms-23-06898-s001.zip › Supplementary Materials 040522.pdf]

Supplementary Materials

Transcriptional landscape of *BRAF* wild type metastatic melanoma

Elena Lastraioli[1*], Federico Alessandro Ruffinatti[2*], Giacomo Bagni[1], Luca Visentin[2], Francesco di Costanzo[3], Luca Munaron[2], Annarosa Arcangeli[1,4§]

[1]Department of Experimental and Clinical Medicine, University of Florence, Viale GB Morgagni 50, 50134 Florence, Italy; [2]Department of Life Sciences and Systems Biology, University of Torino, Via Accademia Albertina 13, 10123 Torino, Italy; [3]Medical Oncology Unit, Azienda Ospedaliero-Universitaria Careggi, Largo Brambilla 3, 50134 Florence, Italy; [4]Complex Dynamics Study Center (CSDC), University of Florence.

*Equally contributed to this paper

[§] Correspondence to:

Annarosa Arcangeli, M.D., Ph.D.

Department of Clinical and Experimental Medicine

Viale GB Morgagni, 50

50134 Florence, Italy

Phone: +39 055 2751285

Fax: +39 055 2751281

e-mail: annarosa.arcangeli@unifi.it

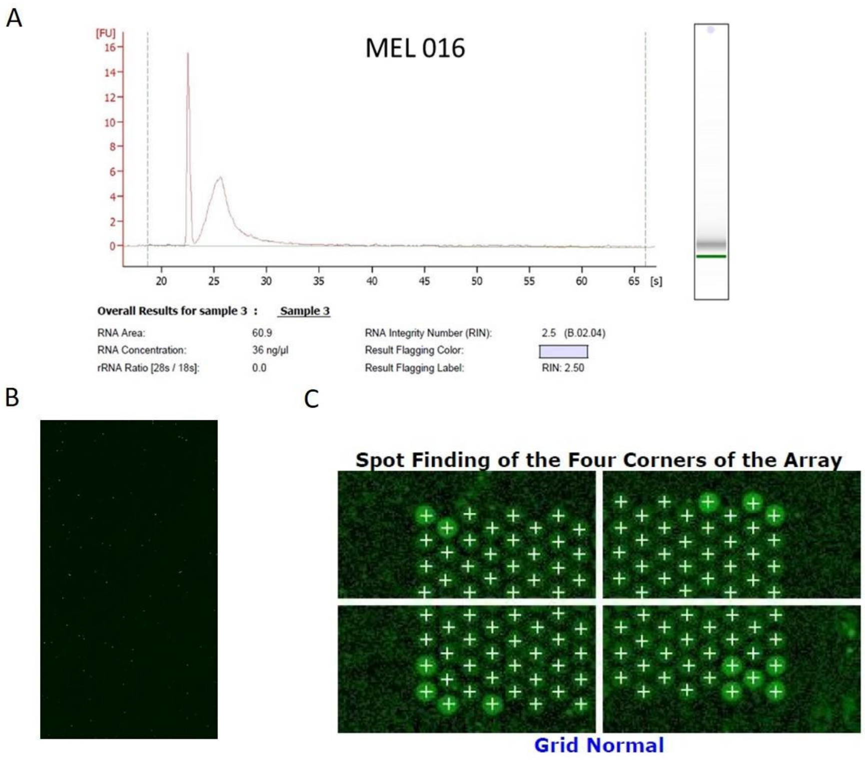

Figure S1. Evaluation of RNA quality and microarray hybridisation. A) *Agilent 2100 Bioanalyzer analysis of a representative specimen showing RNA Integrity Number (RIN = 2.5) consistent with the values generally expected for paraffin-embedded tissues.* ***B)*** *Representative scanning of a microarray slide after hybridisation.* ***C)*** *Magnification of the four corners of the slide in B showing reference probes for grid positioning.*

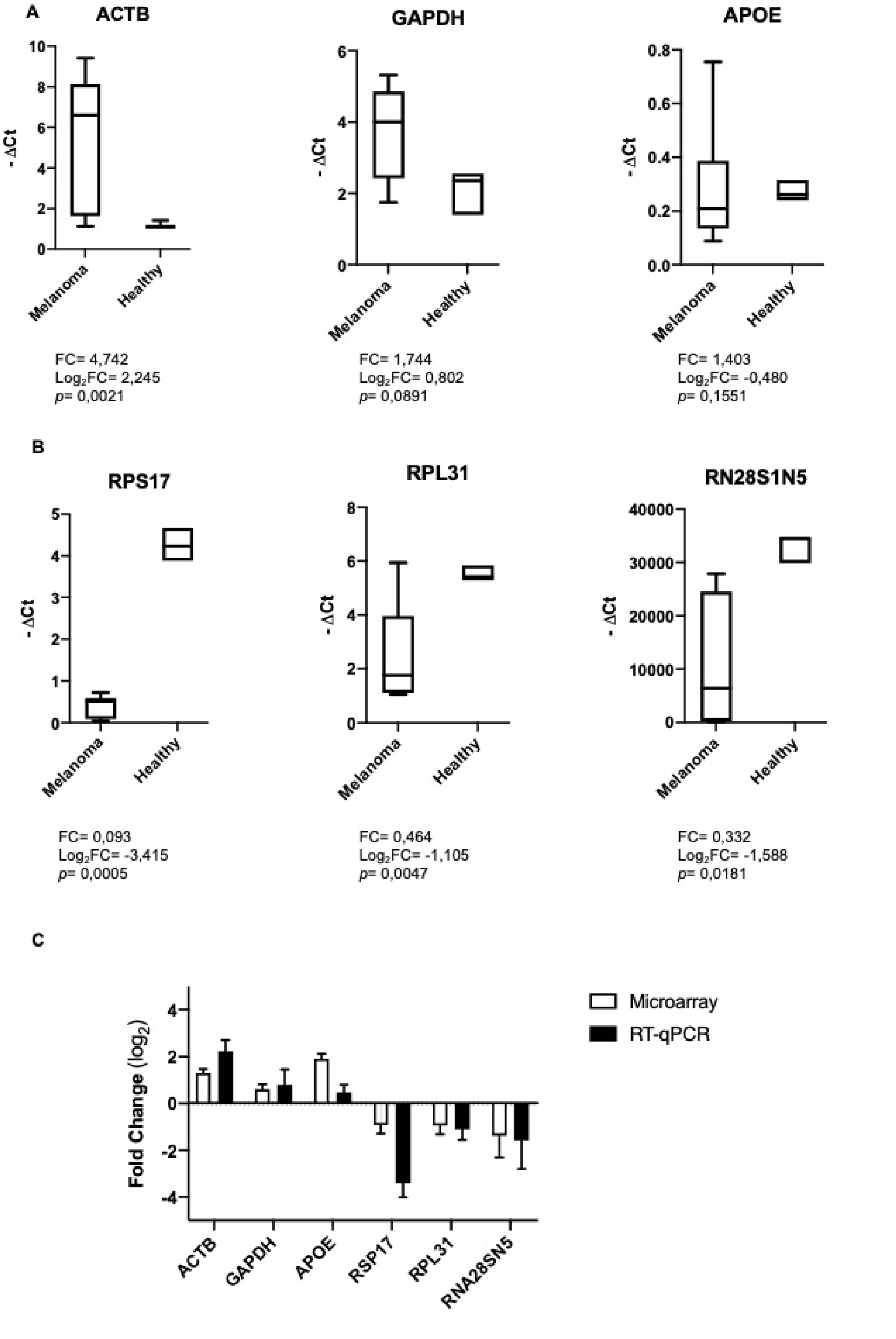

Figure S2. Microarray validation results. A) *RT-qPCR results for a selection of three DEGs up-regulated in melanoma compared to healthy control, using a sample subset (n = 4) from microarray experiment.* ***B)*** *RT-qPCR results for a selection of three DEGs down-regulated in melanoma compared to healthy control, using a sample subset (n = 4) from microarray experiment. Data presented as median and interquartile range (IQR). Ct, threshold cycle; Fold Change ΔΔCt method of relative quantification; p =* p*-value (Mann-Whitney* U*-test).* ***C)*** *Relative expression fold change (log_2) values comparison between microarray and PCR. Data presented as mean ± SEM.*

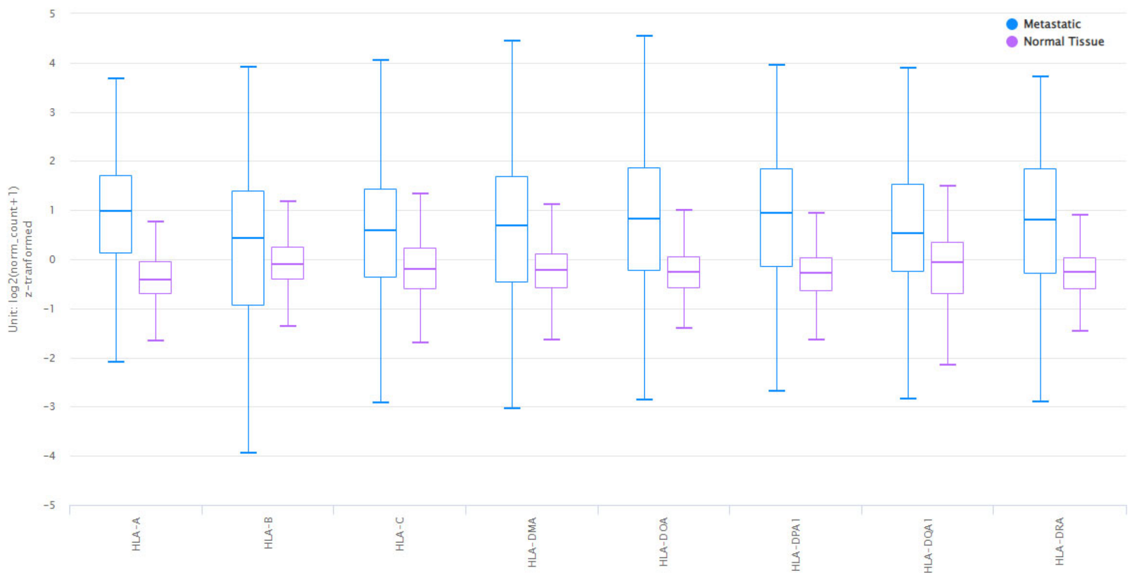

Figure S3. MHC gene expression from TCGA/GTEx databases for metastatic melanoma samples and normal tissues. *A systematic overexpression of all the MHC class I genes (HLA-A, HLA-B, HLA-C), as well as MHC class II (HLA-DMA, HLA-DOA, HLA-DPA1, HLA-DQA1, HLA-DRA) could be confirmed, comparing BRAF-wild type metastatic melanoma samples with healthy skin tissue. Xena browser was used to filter TCGA samples and keep only data from Skin Cutaneous Melanoma studies, of metastatic type, and with no mutations in BRAF gene. The final cohort featured 179 tumour samples from TCGA and 557 healthy samples from GTEx, for a total sample size of n = 736.*
